# Trends and inequalities in physical fitness and BMI among Chinese college students: an analysis of national surveys between 2019 and 2023

**DOI:** 10.3389/fpubh.2026.1814894

**Published:** 2026-04-20

**Authors:** Shucun Sun, Anle Cheng

**Affiliations:** 1 School of Education, Fuyang Normal University, Fuyang, China; 2 School of Urban Planning and Design, Peking University, Shenzhen, China

**Keywords:** BMI, college students, Physical Fitness, Regional inequality, trend analysis

## Abstract

**Background:**

Physical fitness and BMI among Chinese college students have undergone significant changes in recent years amid rapid socioeconomic transitions. Understanding these temporal patterns is essential for guiding effective health promotion strategies.

**Methods:**

Data were extracted from five cycles of the Exercise and physical health dataset of college students (2019–2023), including 58,892 college students aged 16–21. A standardized Physical Fitness Index (PFI) was constructed from six components: vital capacity (FVC), standing long jump (SLJ), sit-and -reach (SAR), muscle strength (MS), 50 m sprint (SR), and 800/1,000 m run (ER). Descriptive analyses evaluated annual, regional, and socioeconomic variations. Restricted cubic spline (RCS) regression models were conducted to assess the nonlinear relationship between BMI and PFI.

**Results:**

Between 2019 and 2023, the prevalence of thinness decreased from 13% to 8.3%, while overweight and obesity increased from 21.5% to 28.6%. The overall median PFI declined from −0.1 (−2.2, 2.2) in 2019 to −2.2 (−4.4, 0.1) in 2022, followed by a recovery to −1.0 (−3.2, 1.4) in 2023. The lowest PFI values were observed among obese students (−4.9 in 2022). Eastern provinces showed higher baseline PFI but smaller declines, while western regions experienced sharper decreases (>1.0 SD decline). BMI demonstrated an inverted–U relationship with PFI, showing a positive association within 18–25 kg/m^2^ and a negative association outside this interval.

**Conclusion:**

From 2019 to 2023, Chinese college students showed declining fitness and rising obesity, with partial recovery. Marked regional inequalities persist, and a nonlinear BMI–PFI pattern indicates that maintaining a moderate BMI is optimal for overall fitness.

## Introduction

Over the past decade, the physical health of Chinese youth has undergone profound transformation. In alignment with the Healthy China 2030 blueprint, the university years constitute a pivotal stage for the development of physical function and lifestyle habits (1). Thus, changes in college students' physical fitness are of substantial public health importance, offering valuable insight into national health trends and informing the optimization of physical and health education policies (2, 3).

Against this backdrop, the relationship between body mass index (BMI) and physical fitness (PFI) among adolescents has attracted growing scholarly attention (4, 5). Emerging evidence suggests that the association between BMI and PFI is not linear but rather exhibits a complex curvilinear pattern. A large-scale national study involving 30,497 college students aged 19–22 found a significant nonlinear association between BMI and PFI: both underweight and overweight/obese students demonstrated markedly lower overall fitness compared with those of normal weight (6). Furthermore, BMI has been widely recognized as a key covariate in studies of adolescent health. For instance, one study identified a significant negative association between BMI and cardiorespiratory fitness, suggesting that increased BMI is a major risk factor for reduced cardiopulmonary health (7). In addition, body image dissatisfaction associated with higher BMI has been linked to elevated risks of depression among adolescents (8). Finally, BMI displays distinct curvilinear relationships with various components of physical fitness, including standing long jump and grip strength (9). Collectively, these findings underscore that BMI serves as a robust indicator of adolescents' physical fitness and a valuable predictor of overall health status (10).

Previous studies have consistently shown that although the nutritional status of Chinese adolescents has steadily improved, their physical fitness levels have exhibited a persistent decline (11, 12). This deterioration in fitness is not only linked to reduced physical activity but also reflects the combined influences of declining sports participation and lifestyle changes. Meanwhile, increased reliance on food delivery and extended sedentary study time contributed to severe imbalances between energy intake and expenditure, making rising obesity rates a concern on university campuses (13). Importantly, given China's vast geographic span and substantial regional heterogeneity in socioeconomic development, both BMI distribution and physical fitness levels may vary considerably across regions (5). Ignoring such spatial variation may limit the generalizability of findings.

Although several regional studies have documented temporary fluctuations in students' physical fitness and health status, these investigations were often limited by small sample sizes, narrow geographic coverage, and short observation periods (14–17). These limitations restrict the ability to simultaneously examine nationwide patterns, regional disparities, and complex BMI–fitness relationships. Consequently, small sample sizes and narrow geographic coverage hinder the robust identification of spatial heterogeneity and non-linear BMI–fitness associations on a national scale, particularly in the post-pandemic era (18).

In response to both practical and academic imperatives, this study draws on national physical fitness monitoring data of Chinese college students from 2019 to 2023 to systematically assess the temporal trends, regional disparities, and interrelations in students' physical fitness and nutritional status. To better capture potential nonlinear associations, BMI was treated as a continuous variable in the analysis rather than relying solely on categorical classifications. Specifically, the study aims to address three key questions: (1) How did the overall levels of physical fitness and nutritional status among Chinese college students change from 2019 to 2023? (2) Do these changes exhibit regional variations? and (3) What are the characteristic associations between BMI and physical fitness indicators among college students? By answering these questions, the study seeks to provide robust empirical evidence to inform future interventions in university health promotion and the refinement of national health policies.

## Methods

### Study design and participants

This study is based on the *Exercise and physical health dataset of college students (2014–2023)*, jointly developed and maintained by Tsinghua University and the National Population Health Data Archive (PHDA). The dataset provides a standardized and traceable data resource for nationwide research on college students' physical fitness. All data were collected in accordance with the *National Student Physical Fitness Standards (Revised 2014)*, ensuring scientific measurement procedures, reliable instruments, and standardized post-collection data cleaning and integration.

For the present analysis, we utilized repeated cross-sectional data from five consecutive survey waves between 2019 and 2023. We focused on college students originating from 31 provinces, autonomous regions, and municipalities in mainland China. Data from Hong Kong, Macao, and Taiwan were excluded. The final analytic sample consisted of 58,892 participants. Data usage strictly adhered to the PHDA Data Use Agreement (CC BY 4.0). All analyses were conducted in compliance with intellectual property and ethical standards, ensuring participant confidentiality and data security.

### Procedures

Physical fitness tests included forced vital capacity (FVC), standing long jump (SLJ), sit-and-reach (SAR), strength tests (pull-ups for males and sit-ups for females), 50–meter sprint (SR), and 800/1,000–meter run (ER), representing six dimensions of physical function: respiratory capacity, lower-limb explosive strength, flexibility, muscular strength, speed, and endurance, respectively. All tests were conducted under standardized conditions and in accordance with the testing protocols issued by the Ministry of Education.

To enable consistent comparisons of physical fitness levels across sex, age, and survey years, the 2019 national dataset of Chinese college students was used as the reference (11, 19). Median values and standard deviations for each fitness indicator were calculated within sex–age subgroups (Supplementary Table S1). Each participant's fitness score was then standardized into a Z-score. In the composite calculation, forced vital capacity, standing long jump, sit-and-reach, and muscular endurance were treated as positive indicators (higher values indicating better performance), whereas the 50-meter sprint and endurance run were treated as negative indicators (lower values indicating better performance). The overall Physical Fitness Index (PFI) was defined as the weighted sum of the standardized scores of these‘ six indicators:


$$\begin{array}{l} PFI=FVC_{z}+SLJ_{z}+SAR_{z}+MS_{z}-SR_{z}-ER_{z} \end{array}$$ (1)

To examine the impact of regional disparities on college students' physical fitness and nutritional status, this study classified participants according to their birthplace (province). China's 31 provincial-level administrative regions were grouped into eastern, central, and western regions to capture variations in economic development and resource environments. Specifically, the eastern region included Beijing, Tianjin, Hebei, Liaoning, Shanghai, Jiangsu, Zhejiang, Fujian, Shandong, Guangdong, and Hainan (11 provinces/municipalities); the central region included Shanxi, Jilin, Heilongjiang, Anhui, Jiangxi, Henan, Hubei, and Hunan (8 provinces); and the western region included Sichuan, Guizhou, Yunnan, Tibet, Shaanxi, Gansu, Qinghai, Ningxia, Xinjiang, Chongqing, Inner Mongolia, and Guangxi (12 provinces/autonomous regions/municipalities). This classification provides a meaningful representation of regional disparities in socioeconomic development levels across China (20).

### Statistical analysis

We first conducted descriptive analyses to summarize the demographic characteristics, nutritional status (classified by BMI), and physical fitness performance of college students across the five survey years (2019–2023). Differences between groups for categorical variables were assessed using the chi-square (χ^2^) test, while differences in continuous variables—given their non-normal distributions—were evaluated using the Kruskal–Wallis rank-sum test. When a significant overall difference was detected, Dunn's *post-hoc* test with Bonferroni correction was subsequently performed for pairwise comparisons between survey years.

To explore the potential non-linear association between BMI and PFI, we employed a restricted cubic spline (RCS) model. Considering the balance between model fit and complexity, the RCS was configured with four knots located at the 5th, 35th, 65th, and 95th percentiles of the BMI distribution. The model is expressed by the following equation:


$$\begin{array}{l} PFI=\beta_{0}+\beta_{1}BMI+\beta_{2}BMI^{\prime }+\beta_{3}BMI^{\prime \prime }+\sum_{i=1}^{n}\gamma_{i}C_{i}+\epsilon \end{array}$$ (2)

Where *BMI* represents the linear component, and *BMI*′ and *BMI*″are the basis functions representing the non-linear components derived from the spline transformation. *C*_*i*_ represents the vector of adjusted covariates, including gender, age, grade, ethnicity, and regions.

The Wald test was performed to evaluate both the overall association and the significance of non-linearity (21). A *P*-value for non-linearity less than 0.05 was considered to indicate a significant non-linear relationship.

## Results

### Basic characteristics of the study population

As shown in Table 1, a total of 58,892 college students aged 16–21 years were included across the five survey years: 11,079 in 2019, 10,970 in 2020, 12,695 in 2021, 11,909 in 2022, and 12,239 in 2023. First-year students accounted for 28.6% (16,831), second-year students 28.0% (16,514), third-year students 25.8% (15,184), and fourth-year students 17.6% (10,363).

**Table 1:** Baseline characteristics of participants Chinese college students from 2019 to 2023.

Variable	2019 *N* = 11,079^1^	2020 *N* = 10,970^1^	2021 *N* = 12,695^1^	2022 *N* = 11,909^1^	2023 *N* = 12,239^1^	*P*-value^2^
Gender						
Boys	7,490 (68%)	7,422 (68%)	8,729 (69%)	8,303 (70%)	8,799 (72%)	< 0.001
Girls	3,589 (32%)	3,548 (32%)	3,966 (31%)	3,606 (30%)	3,440 (28%)	
Grade						
First-year	3,409 (31%)	3,361 (31%)	3,406 (27%)	3,209 (27%)	3,446 (28%)	< 0.001
Second-year	3,386 (31%)	3,299 (30%)	3,482 (27%)	3,156 (27%)	3,191 (26%)	
Third-year	2,793 (25%)	2,993 (27%)	3,269 (26%)	3,087 (26%)	3,042 (25%)	
Fourth-year	1,491 (13%)	1,317 (12%)	2,538 (20%)	2,457 (21%)	2,560 (21%)	
Ethnicity						
Han	9,741 (88%)	9,788 (89%)	11,308 (89%)	10,689 (90%)	11,001 (90%)	< 0.001
Minorities	1,338 (12%)	1,182 (11%)	1,387 (11%)	1,220 (10%)	1,238 (10%)	
Regions						
East	4,504 (41%)	4,525 (41%)	5,166 (41%)	4,771 (40%)	4,921 (40%)	0.008
Middle	2,566 (23%)	2,575 (23%)	3,073 (24%)	3,002 (25%)	3,035 (25%)	
West	4,009 (36%)	3,870 (35%)	4,456 (35%)	4,136 (35%)	4,283 (35%)	
Nutritional status						
Normal	7,202 (65%)	7,060 (64%)	8,123 (64%)	7,436 (62%)	7,689 (63%)	< 0.001
Obesity	609 (5.5%)	719 (6.6%)	906 (7.1%)	954 (8.0%)	1,053 (8.6%)	
Overweight	1,776 (16%)	2,140 (20%)	2,431 (19%)	2,422 (20%)	2,476 (20%)	
Thinness	1,492 (13%)	1,051 (9.6%)	1,235 (9.7%)	1,097 (9.2%)	1,021 (8.3%)	
Age						
16	3,110 (28%)	2,758 (25%)	1,899 (15%)	89 (0.7%)	100 (0.8%)	< 0.001
17	2,548 (23%)	2,950 (27%)	3,775 (30%)	2,626 (22%)	1,216 (9.9%)	
18	3,119 (28%)	2,984 (27%)	4,635 (37%)	4,309 (36%)	3,365 (27%)	
19	2,070 (19%)	1,954 (18%)	2,092 (16%)	2,762 (23%)	2,930 (24%)	
20	196 (1.8%)	283 (2.6%)	245 (1.9%)	1,888 (16%)	2,885 (24%)	
21	36 (0.3%)	41 (0.4%)	49 (0.4%)	235 (2.0%)	1,743 (14%)	
Height (cm)	173 (167, 178)	172 (166, 178)	172 (166, 178)	173 (167, 178)	173 (167, 178)	< 0.001
Weight (kg)	64 (56, 72)	65 (57, 74)	65 (57, 74)	65 (57, 75)	66 (58, 75)	< 0.001
BMI	21.3 (19.5, 23.6)	21.8 (19.9, 24.1)	21.8 (19.9, 24.1)	22.0 (20.0, 24.4)	22.1 (20.1, 24.5)	< 0.001
FVC	3,852 (3,187, 4,475)	4,043 (3,325, 4,672)	3,793 (3,129, 4,379)	3,821 (3,175, 4,395)	3,869 (3,218, 4,438)	< 0.001
SLJ	216 (185, 236)	220 (188, 240)	213 (183, 233)	215 (185, 236)	215 (186, 235)	< 0.001
SAR	13 (8, 18)	14 (9, 18)	15 (11, 20)	14 (9, 18)	15 (10, 19)	< 0.001
MS	12 (3, 36)	8 (1, 34)	10 (3, 35)	8 (1, 31)	10 (2, 32)	< 0.001
SR	7.40 (6.90, 8.30)	7.90 (7.50, 8.90)	7.50 (7.00, 8.30)	7.80 (7.40, 8.80)	7.50 (7.00, 8.20)	< 0.001
ER	240 (225, 258)	248 (231, 270)	248 (230, 271)	256 (236, 280)	254 (234, 278)	< 0.001
PFI	−0.1 (-2.2, 2.2)	−1.1 (-3.3, 1.2)	−0.5 (-2.8, 1.8)	−2.2 (-4.4, 0.1)	−1.0 (-3.2, 1.4)	< 0.001

^1^n (%); Median (Q1, Q3).

^2^Pearson's Chi-squared test; Kruskal-Wallis rank sum test.

We used geographical region (east, middle, and west) as an indicator for economic development, which correspond to developed, intermediately developed, and underdeveloped economic status.

Regarding physical fitness and nutritional status, we observed a decline in the prevalence of thinness from 2019 to 2023, alongside increases in the prevalence of overweight and obesity. Specifically, PFI showed a marked decline from 2019 to 2022, with the median decreasing to −2.2 (interquartile range: −4.4, 0.1), followed by a partial rebound to −1.0 (−3.2, 1.4) in 2023. The Kruskal–Wallis test indicated significant differences across years (p < 0.001), and Dunn's *post-hoc* comparisons correction further confirmed that PFI in 2022 was significantly lower than in all other years (all adjusted *P* < 0.001). Additionally, significant differences were observed among most year-to-year comparisons (all adjusted *P* < 0.001), except between 2020 and 2023 (adjusted *P* = 0.913). Consequently, PFI reached its lowest point in 2022, coinciding with the highest proportion of students classified as overweight or obese. Additionally, notable changes in nutritional status were observed beginning in 2019: the annual proportion of students with normal BMI declined, whereas the proportion of those who were overweight or obese increased over the same period.

### Temporal trends in physical fitness and nutritional status

**Figure 1:**
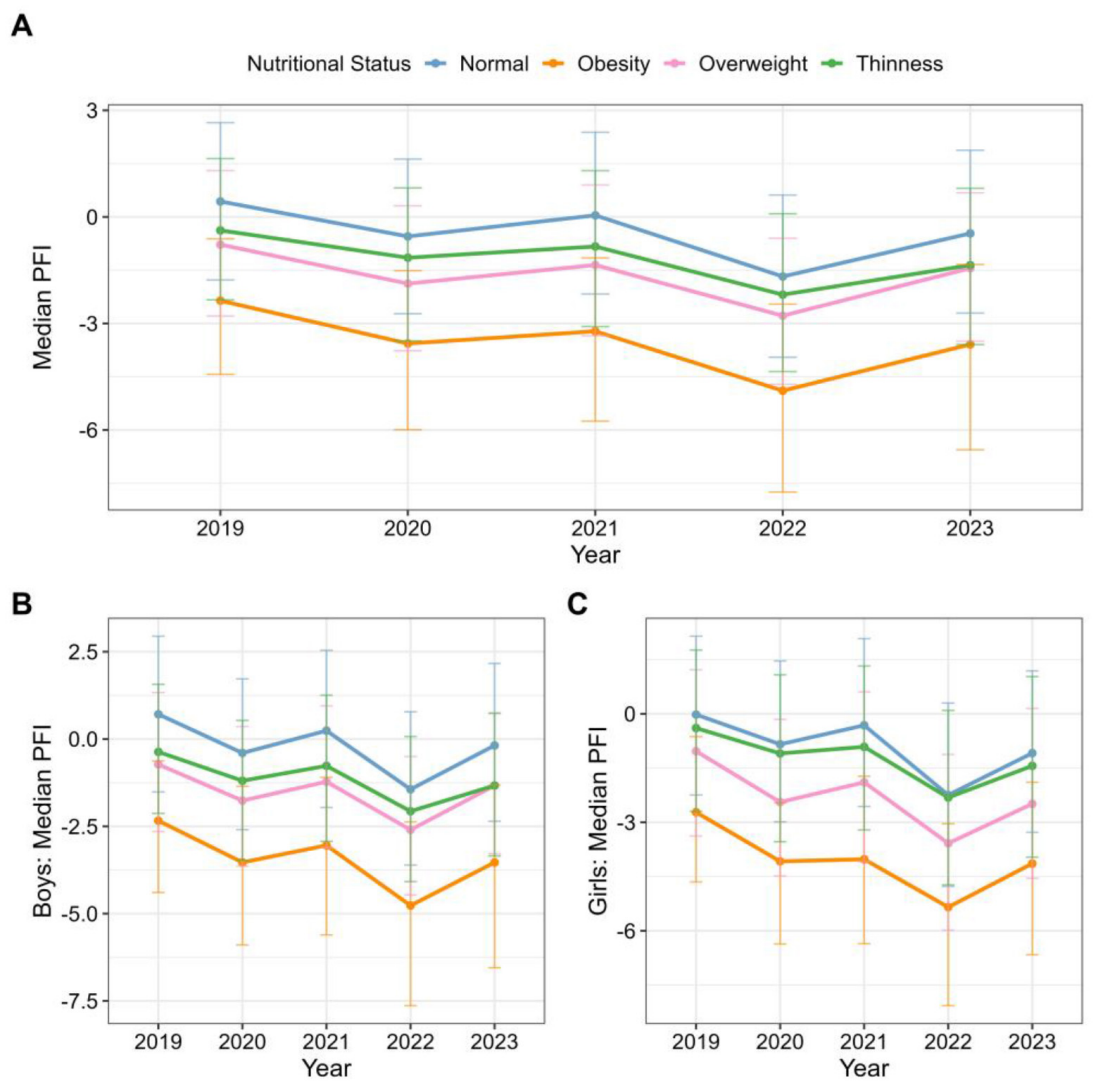
PFI of college students across different nutritional status categories. **A**. Boys. **B**. Girls.

As illustrated in Figure 1A and Supplementary Table S2, the PFI of college students across different nutritional status categories exhibited significant temporal trends from 2019 to 2023. Overall, PFI declined across all BMI groups, with the decrease being most pronounced among overweight and obese students. In the normal-weight group, the median PFI dropped from 0.44 (IQR: −1.77 to 2.65) in 2019 to −1.68 (−3.95 to 0.62) in 2022, before slightly rebounding to −0.46 (−2.71 to 1.88) in 2023. In contrast, the obese group consistently showed the lowest PFI levels throughout the monitoring period, decreasing from −2.36 (−4.43 to −0.62) in 2019 to −4.89 (−7.75 to −2.46) in 2022.

By 2023, all four BMI groups demonstrated some degree of recovery in PFI: the median in the normal-weight group rose from −1.68 to −0.46, in the obese group from −4.89 to −3.59, in the overweight group from −2.78 to −1.44, and in the thinness group from −2.19 to −1.36.

Figures 1B–C and Supplementary Table S3 show that PFI steadily declined across all BMI groups for both males and females from 2019 to 2022, reaching the lowest values of the period in 2022. Among boys, the largest declines were observed in the obese group (−4.77) and the overweight group (−2.60). Grils experienced more pronounced decreases, with the obese group dropping from −2.72 to −5.34 and the overweight group from −1.03 to −3.58.

By 2023, PFI showed varying degrees of recovery across all sex-specific groups. In boys, the greatest rebounds occurred in the overweight and normal-weight groups, while the thickness group exhibited the smallest recovery. In girls, the largest improvements were observed in the obese and normal-weight groups, with the thickness group again showing the least recovery.

**Figure 2:**
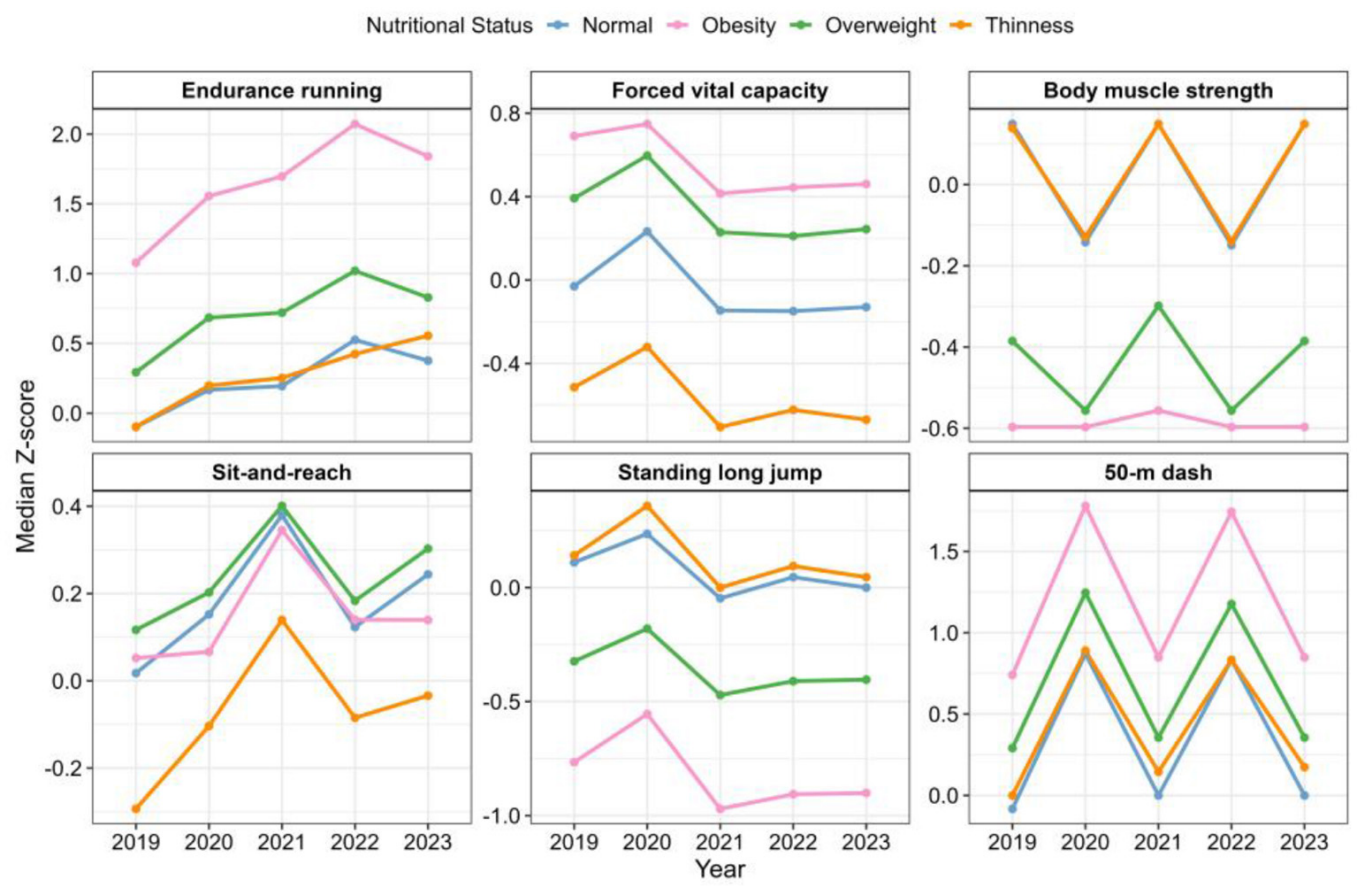
Trends in physical fitness components by nutritional status, 2019–2023.

As shown in Figure 2 and Supplementary Table S4, analyses for each individual physical fitness indicator reveal distinct patterns across nutritional status groups. Overall, students in the normal-weight group had median Z-scores close to or slightly above zero for FVC, SAR, MS, and SLJ, indicating average levels of cardiorespiratory function, strength, and flexibility. For the negative indicators, SR and ER, median Z-scores were slightly below zero, reflecting relatively better sprinting speed and endurance performance.

In contrast, boys in the obese group exhibited markedly higher Z-scores for the negative indicators ER and SR (ER median increased from 1.08 in 2019 to 1.84 in 2023; SR from 0.74 to 0.85), indicating substantially lower running speed and endurance compared with other groups. Strength-related indicators such as MS and SLJ were negative for the obese group, corroborating the prevalence of “obesity-related sarcopenia” among adolescents (22). Students in the overweight group showed intermediate values between the normal-weight and obese groups, with some cardiorespiratory and strength indicators slightly below normal-weight students but clearly better than those of the obese group. Boys in the thickness group had lower median values for cardiorespiratory endurance indicators (FVC, ER), while MS and SLJ were near or slightly above normal, suggesting relatively balanced strength but insufficient endurance.

Longitudinal trends indicate significant differences in the trajectories of PFI among nutritional status groups. For ER, Z-scores increased year by year, especially in the obese group. The median ER for obese boys rose sharply from 1.08 in 2019 to a peak of 2.07 in 2022, and remained elevated at 1.84 in 2023, clearly indicating a rapid decline in aerobic endurance relative to age-matched peers. SR exhibited the largest fluctuations among all fitness indicators, reflecting the considerable influence of individual differences.

Additionally, declines in FVC and SLJ were observed across groups. Conversely, SAR increased significantly, suggesting that agility and coordination continued to improve within this population.

### Spatial inequalities in physical fitness and nutritional status

**Figure 3:**
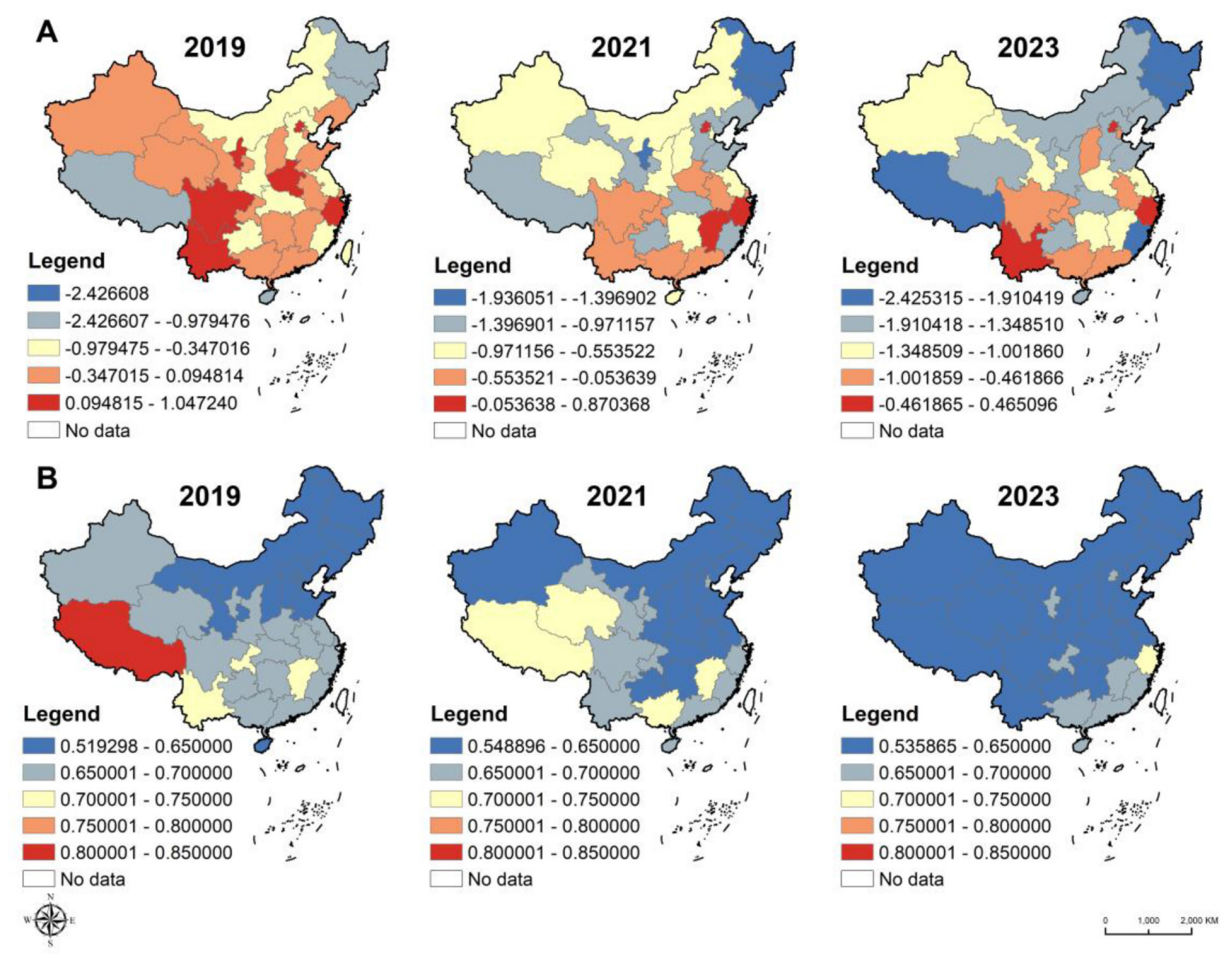
Spatial inequalities in physical fitness and nutritional status. **A**. Spatial distribution of median PFI. **B**. Spatial distribution of the proportion of college students with normal BMI.

As shown in Figure 3A and Supplementary Table S5, the province-level trends indicate that from 2019 to 2023, the overall PFI of college students declined continuously across all provinces, though the magnitude of change varied considerably by region. In general, students born in eastern provinces started with higher fitness levels. For instance, in 2019, the PFI values for students from Beijing, Shanghai, and Zhejiang were 1.05, −0.10, and 0.64, respectively, but all showed notable decreases by 2023, dropping to 0.47, −1.13, and −0.20. In contrast, students from central and western provinces experienced steeper declines — provinces such as Ningxia, Guizhou, Xinjiang, and Heilongjiang saw reductions exceeding 1 standardized score unit, indicating a more pronounced deterioration in physical fitness levels.

Overall, while eastern provinces maintained higher starting levels with a more gradual decline, central and western regions began at lower baselines and experienced sharper decreases, leading to a widening regional disparity in college students' physical fitness.

Figure 3B and Supplementary Table S6 illustrate that from 2019 to 2023, the proportion of college students with a *normal nutritional status* nationwide showed a slight overall decline. Most regions reached relatively high levels in 2019, followed by gradual decreases over subsequent years. For instance, the proportion of students with normal weight dropped from 69.6% to 62.6% in Shanghai, from 70.7% to 62.2% in Yunnan, and from 67.0% to 64.3% in Sichuan. This downward trend indicates a growing prevalence of abnormal weight conditions among college students, likely reflecting recent lifestyle and dietary changes.

### Associations between physical fitness and nutritional status

**Figure 4:**
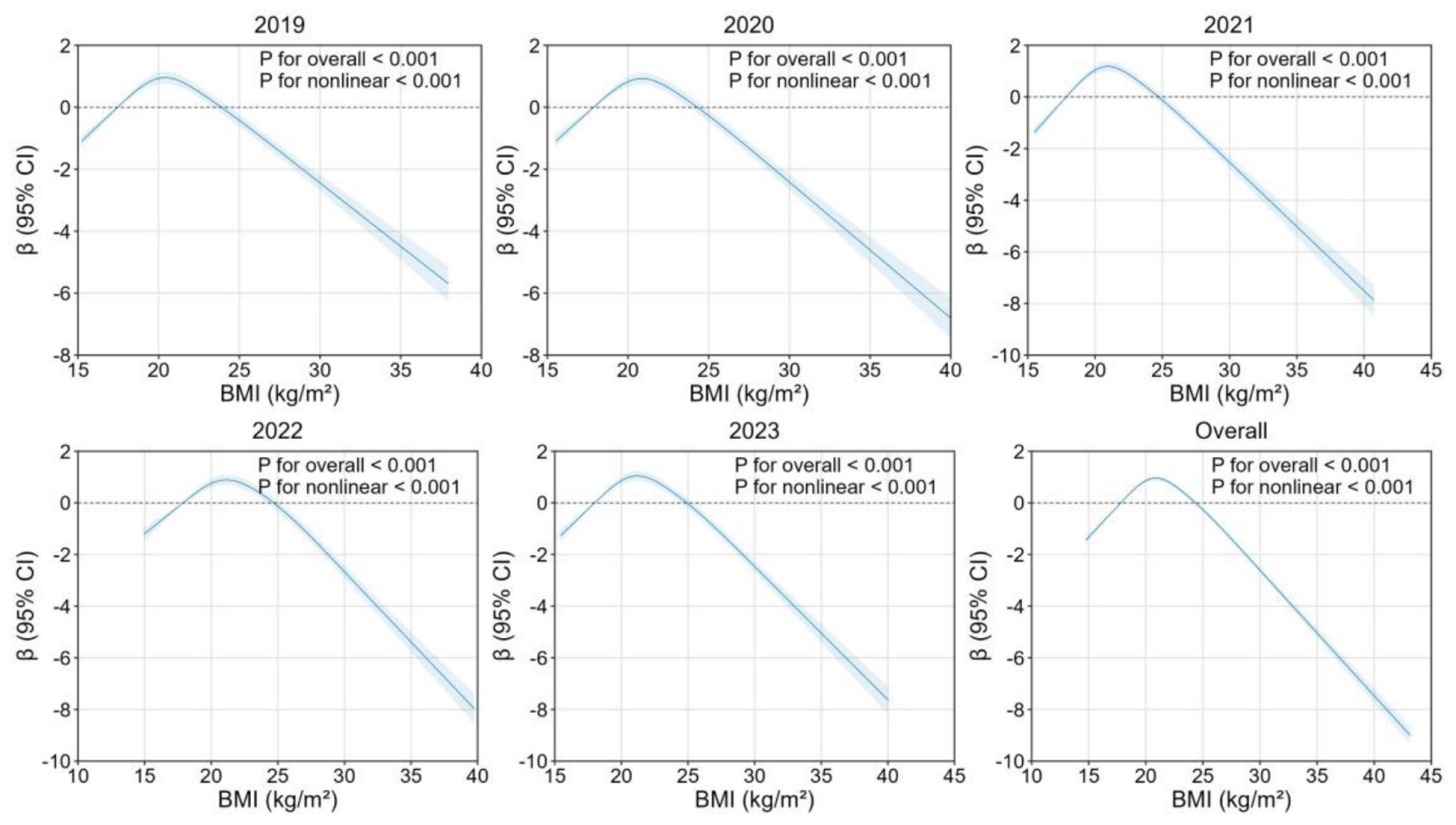
Restricted cubic spline plots of the association between BMI and PFI (2019–2023).

Figure 4 and Supplementary Tables S7 present quadratic regression analyses, systematically illustrating the nonlinear relationship between BMI and the PFI among Chinese college students. The restricted cubic spline (RCS) models revealed a consistent inverted U-shaped relationship between BMI and the PFI across all studied years (2019–2023) and in the overall population (all P for non-linearity < 0.001; P for overall association < 0.001).

Specifically, the estimated coefficients (β) were positive only when BMI ranged from approximately 18 to 25 kg/m^2^, indicating that within this interval, BMI exerts a favorable influence on PFI compared to the reference. Conversely, when BMI fell below 18 kg/m^2^ (extreme underweight) or exceeded 25 kg/m^2^ (overweight and obesity), the coefficients dropped below the zero threshold (β < 0), signifying a detrimental impact on PFI. The non-linear pattern remained remarkably stable over the five-year period.

**Figure 5:**
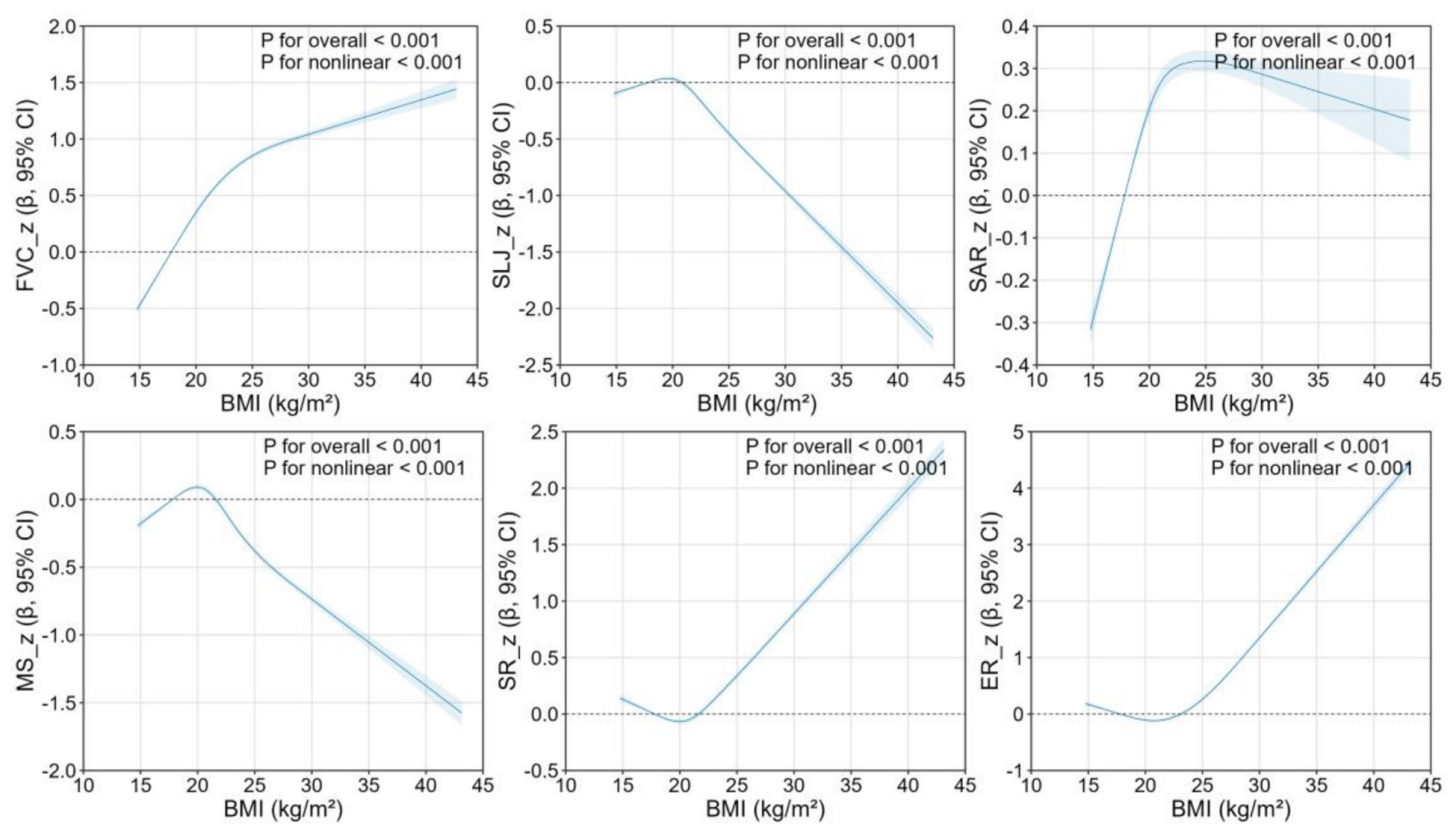
Nonlinear associations between BMI and individual physical fitness components among Chinese college students.

Figure 5 and Supplementary Table S8 further present regression analyses between BMI and individual physical fitness indicators among Chinese college students. Restricted cubic spline analyses demonstrated significant associations between BMI and all physical fitness indicators, with distinct nonlinear patterns observed across different measures.

For forced vital capacity (FVC_z), BMI exhibited a threshold effect. The regression coefficient crossed the zero-reference line at approximately BMI 18 kg/m^2^, beyond which it remained consistently positive. This suggests that a higher BMI is a strong promoter of absolute lung volume.

In contrast, standing long jump (SLJ_z) and muscular strength (MS_z) followed a strict “optimal window” pattern. For SLJ_z, the positive effect (β>0) was only observed in a narrow interval around BMI 18–21 kg/m^2^, reaching its peak at approximately 20 kg/m^2^. Beyond a BMI of 22 kg/m^2^, the coefficient plummeted deep into the negative zone. MS_z showed a similar parabolic trend, with its positive effect window also confined to the normal BMI range.

Sit-and-reach (SAR_z) demonstrated a broader positive range. The effect of BMI turned positive at BMI 18 kg/m^2^ and remained above the zero line across the entire studied range, although the magnitude of this positive association began to slightly decline after BMI 25 kg/m^2^.

Interestingly, the 50 m sprint (SR_z) and endurance running (ER_z) exhibited a ‘U-shaped' coefficient curve. While the effect was near zero in the 25 kg/m^2^ range, the coefficients rose sharply at higher BMI levels. This suggests that higher BMI values are associated with significantly higher z-scores, reflecting a marked decline in speed and aerobic capacity among overweight and obese students.

## Discussion

Using five consecutive waves of exercise and physical health dataset of college students data (2019–2023), this study provides a comprehensive assessment of the physical fitness and nutritional status of nearly 60,000 Chinese college students across 31 provinces. Overall, the physical fitness level of college students showed a marked decline over the study period, accompanied by a steady increase in the prevalence of overweight and obesity. These results are consistent with previous research suggesting that social isolation and unhealthy dietary habits may have led to decreased physical activity and poor nutrition, thereby adversely affecting youth fitness (23–25).

From a spatial perspective, this study reveals a clear inequality pattern in fitness and nutritional status. Students from eastern provinces started at higher fitness levels and experienced milder declines, whereas those from central–western and northern regions exhibited lower baselines and steeper downward trends (26). Meanwhile, the proportion of students with “normal” nutritional status showed a gradual national decline, though several central–western and southern provinces maintained relatively higher levels. In contrast, northeastern and northern provinces had lower proportions, reflecting more pronounced weight-related issues (27).

At the individual level, the nonlinear association between BMI and PFI was evident across all years and demographic subgroups (28–30). The consistently inverted U-shaped relationship indicates an optimal BMI range of approximately 18–25 kg/m^2^, beyond which physical performance declines. When analyzing individual fitness components, BMI–fitness relationships displayed clear heterogeneity. FVC followed an inverted U-shaped pattern (31, 32); SLJ and MS declined with increasing BMI (33); SAR showed a similar inverted U-shape; SR and ER performance declined sharply for overweight and obese students (34). The limited temporal variation in turning points suggests that the nonlinear influence of BMI on fitness components has remained stable over time (35). This multidimensional heterogeneity reflects underlying physiological mechanisms. Indicators of pulmonary function, strength, and flexibility perform best within a moderate BMI range—likely due to optimal muscle mass, fat distribution, and joint mobility—whereas extreme BMI values impair speed and endurance, either through insufficient energy reserves or excessive body burden (36–38).

These findings carry important public health and policy implications. First, universities should prioritize interventions for vulnerable groups—particularly thickness and obese students—by implementing tailored exercise prescriptions and nutritional programs to optimize BMI distribution and enhance overall fitness. Second, individualized intervention strategies should consider the heterogeneity of BMI–fitness relationships: aerobic training may benefit underweight students with poor endurance, whereas strength and flexibility training may improve performance among high-BMI students.

Several limitations should be acknowledged. First, this study focuses exclusively on college students and excludes employed youth and populations from Hong Kong, Macao, and Taiwan, which may limit the generalizability of findings. Second, the cross-sectional design allows examination of associations but not causal relationships between BMI and fitness indicators. Future longitudinal or cohort studies are warranted to establish causality. Third, behavioral determinants such as diet, physical activity habits, and lifestyle factors were not included; incorporating these variables in future research would deepen understanding of the mechanisms linking BMI and physical fitness. Finally, future studies should utilize multilevel models with regional random effects to more rigorously investigate the interplay between individual characteristics and macro-level socioeconomic factors.

## Data Availability

The datasets presented in this article are not readily available because data were accessed under a data use agreement that prohibits redistribution. Requests to access the datasets should be directed to https://www.ncmi.cn.

## References

[1] Chen P Li F Harmer P. Healthy China 2030: moving from blueprint to action with a new focus on public health. Lancet Public Health. (2019) 4:e447. doi: 10.1016/S2468-2667(19)30160-4 31493840

[2] Kyröläinen H Santtila M Nindl BC Vasankari T. Vasankari T. Physical fitness profiles of young men: associations between physical fitness, obesity and health. Sports Med. (2010) 40:907–20. doi: 10.2165/11536570-000000000-00000 20942508

[3] Duchin O Marin C Mora-Plazas M Villamor E. Villamor E. Maternal body image dissatisfaction and BMI change in school-age children. Public Health Nutr. (2016) 19:287–92. doi: 10.1017/S1368980015001317 25925877 PMC10271033

[4] Tian Y Jiang C Wang M Cai R Zhang Y He Z . BMI, leisure-time physical activity, and physical fitness in adults in China: results from a series of national surveys, 2000–14. Lancet Diabetes & endocrinology. (2016) 4:487–97. doi: 10.1016/S2213-8587(16)00081-4 27133172

[5] Dong Y Jan C Ma Y Dong B Zou Z Yang Y . Economic development and the nutritional status of Chinese school-aged children and adolescents from 1995 to 2014: an analysis of five successive national surveys. Lancet Diabetes & endocrinology. (2019) 7:288–99. doi: 10.1016/S2213-8587(19)30075-0 30902266

[6] Li S Cao H Liu H Hu Y Liu J. Hu Y, Liu J. Relationship between body mass index and physical fitness index in Chinese college students: Results from a cross-sectional survey. Am J Hum Biol. (2023) 35:e23854. doi: 10.1002/ajhb.23854 36576438

[7] Billingsley HE Del Buono MG Canada JM Kim Y Damonte JI Trankle CR . Sarcopenic obesity is associated with reduced cardiorespiratory fitness compared with nonsarcopenic obesity in patients with heart failure with reduced ejection fraction. Circ Heart Fail. (2022) 15:e009518. doi: 10.1161/CIRCHEARTFAILURE.122.009518 36098058 PMC9588574

[8] Blundell E De Stavola BL Kellock MD Kelly Y Lewis G McMunn A . Longitudinal pathways between childhood BMI, body dissatisfaction, and adolescent depression: an observational study using the UK Millenium Cohort Study. Lancet Psychiatry. (2024) 11:47–55. doi: 10.1016/S2215-0366(23)00365-6 38101872 PMC11139652

[9] Wang X Wang H Yuan X Cai S Huang Y Song Y . Imbalance between muscle strength development and weight gain in children and young adults in China: serial cross-sectional evidence from 1.33 million students from five successive national surveys between 2000 and 2019. Lancet Reg Health West Pac. (2025) 61:101640. doi: 10.1016/j.lanwpc.2025.101640 40747129 PMC12311536

[10] Katzmarzyk PT. Expanding our understanding of the global impact of physical inactivity. Lancet Global Health. (2023) 11:e2–3. doi: 10.1016/S2214-109X(22)00482-X 36480932

[11] Cai S Zhang Y Chen Z Liu Y Dang J Li J . Secular trends in physical fitness and cardiovascular risks among Chinese college students: an analysis of five successive national surveys between 2000 and 2019. Lancet Reg Health West Pac. (2025) 58; doi: 10.1016/j.lanwpc.2025.101560 40336579 PMC12053983

[12] Dong X Huang F Starratt G Yang Z. Yang Z. Trend in health-related physical fitness for Chinese male first-year college students: 2013–2019. Front Public Health. (2023) 11:984511. doi: 10.3389/fpubh.2023.984511 36935701 PMC10014614

[13] Wang X Yang X. Juzaily bin Mohd Nasiruddin N, et al. Social support and physical activity in college and university students: a meta-analysis. Health Educ Behav. (2024) 51:533–43. doi: 10.1177/10901981231216735 38305027

[14] Ye X Zhang J Liu H Zheng X Ye W Fu W . Changes of college students' psychological stress during the COVID-19 pandemic in China: A two-wave repeated survey. J Health Psychol. (2025) 30:736–48. doi: 10.1177/13591053241246620 38660775

[15] Zhou C Zhang A. Longitudinal study on the impact of public health event control measures on physical fitness among Chinese adolescents: a southern city perspective. BMC Public Health. (2024) 24:3615. doi: 10.1186/s12889-024-20751-y 39736530 PMC11684066

[16] Jiang Q Huang X Wang Z Dai X Li R Cui D. Regional differences of physical fitness and overweight and obesity prevalence among college students before and after COVID-19 pandemic since the “double first-class” initiative in China. Front Public Health. (2023) 11:125(2270) doi: 10.3389/fpubh.2023.1252270 38249415 PMC10796554

[17] Bi C Zhang F Gu Y Song Y Cai X. Song Y, Cai X. Secular trend in the physical fitness of Xinjiang children and adolescents between 1985 and 2014. Int J Environ Res Public Health. (2020) 17:2195. doi: 10.3390/ijerph17072195 32218289 PMC7177309

[18] Huang X Zeng L Wang Y Liang H Xu X White M. From neighborhoods to streetscapes: Pandemic-era shifts in built-environment effects on pedestrian mobility. Cities. (2026) 170:106685. doi: 10.1016/j.cities.2025.106685

[19] Huang Y-C Malina RM BMI. and health-related physical fitness in Taiwanese youth 9-18 years. Med Sci Sports Exerc. (2007) 39:701–8. doi: 10.1249/mss.0b013e31802f0512 17414809

[20] Wang M Wen X Zhang Y Jiang C Wang F. Is economic environment associated with the physical activity levels and obesity in Chinese adults? A cross-sectional study of 30 regions in China. BMC Public Health. (2017) 17:701. doi: 10.1186/s12889-017-4699-4 28899367 PMC5596845

[21] Wei C Zhang G. Association between body roundness index (BRI) and gallstones: results of the 2017-2020 national health and nutrition examination survey (NHANES). BMC Gastroenterol. (2024) 24:192. doi: 10.1186/s12876-024-03280-1 38840060 PMC11155175

[22] Münte E Zhang X Khurana A Hartmann P. Hartmann P. Prevalence of Extremely Severe Obesity and Metabolic Dysfunction Among US Children and Adolescents. JAMA Netw Open. (2025) 8:e2521170. doi: 10.1001/jamanetworkopen.2025.21170 40668581 PMC12268495

[23] Gotlib IH Miller JG Borchers LR Coury SM Costello LA Garcia JM . Effects of the COVID-19 Pandemic on Mental Health and Brain Maturation in Adolescents: Implications for Analyzing Longitudinal Data. Biol Psychiatry Glob Open Sci. (2022) 3:912–8. doi: 10.1016/j.bpsgos.2022.11.002 36471743 PMC9713854

[24] Jensen FK Gribsholt SB Schwartz S Andersen AL Bruun JM. Body Mass Index in Children Before, During, and After the COVID-19 Pandemic. JAMA Netw Open. (2025) 8:e251(9528) doi: 10.1001/jamanetworkopen.2025.19528 40632536 PMC12242698

[25] Anderson KN Swedo EA Trinh E Ray CM Krause KH Verlenden JV . Adverse Childhood Experiences During the COVID-19 Pandemic and Associations with Poor Mental Health and Suicidal Behaviors Among High School Students - Adolescent Behaviors and Experiences Survey, United States, January-June 2021. MMWR Morb Mortal Wkly Rep. (2022) 71:1301–5. doi: 10.15585/mmwr.mm7141a2 36227769 PMC9575476

[26] Xiao Q Xiao H. Spatial non-equilibrium and distribution dynamic evolution of the development level of national physical fitness in China's provinces. PLoS ONE. (2024) 19:e0287806. doi: 10.1371/journal.pone.0287806 39110755 PMC11305533

[27] Song X Zhou B Baird S Lu C Ezzati M Chen L . Trends and inequalities in thinness and obesity among Chinese children and adolescents: evidence from seven national school surveys between 1985 and 2019. Lancet Public Health. (2024) 9:e1025–e36. doi: 10.1016/S2468-2667(24)00211-1 39481418 PMC7616785

[28] Chen G Chen J Liu J Hu Y Liu Y. Hu Y, Liu Y. Relationship between body mass index and physical fitness of children and adolescents in Xinjiang, China: a cross-sectional study. BMC Public Health. (2022) 22:1680. doi: 10.1186/s12889-022-14089-6 36064657 PMC9442906

[29] Zhao D Chen X Zhang A Wang C Wang Y He J . An investigation of the relationship and pathways of influence between body mass index, motor coordination, and health-related physical fitness index in preschool children. Front Public Health. (2025) 13:1585768. doi: 10.3389/fpubh.2025.1585768 40746676 PMC12310465

[30] Zhang M Schumann M Huang T Törmäkangas T Cheng S. Törmäkangas T, Cheng S. Normal weight obesity and physical fitness in Chinese university students: an overlooked association. BMC Public Health. (2018) 18:1334. doi: 10.1186/s12889-018-6238-3 30509225 PMC6278052

[31] Guo T Shen S Yang S Yang F. Yang F. The relationship between BMI and physical fitness among 7451 college freshmen: a cross-sectional study in Beijing, China. Front Physiol. (2024) 15:1435157. doi: 10.3389/fphys.2024.1435157 39473612 PMC11519527

[32] Zhang H Sun L Yu Y Xin H Wu L Yang F . The associations between body composition and vital capacity index of medical students in Shenyang of China: a cross-sectional survey. BMC Pulm Med. (2022) 22:373. doi: 10.1186/s12890-022-02176-8 36184644 PMC9526916

[33] Manzano-Carrasco S Garcia-Unanue J Haapala EA Felipe JL Gallardo L Lopez-Fernandez J . Relationships of BMI, muscle-to-fat ratio, and handgrip strength-to-BMI ratio to physical fitness in Spanish children and adolescents. Eur J Pediatr. (2023) 182:2345–57. doi: 10.1007/s00431-023-04887-4 36881145 PMC9989582

[34] Buresh R. Should body size categories be more common in endurance running events? Curr Sports Med Rep. (2018) 17:159–62. doi: 10.1249/JSR.0000000000000481 29738321

[35] Wu J Shao Y Hu J Zhao X. Zhao X. Exploring the link between BMI and physical fitness in Sichuan's ethnic minority primary school students: insights from quantile regression analysis. BMC Pediatr. (2025) 25:478. doi: 10.1186/s12887-025-05647-z 40596982 PMC12220373

[36] Qin G Qin Y Liu B. Association between BMI and health-related physical fitness: a cross-sectional study in Chinese high school students. Front Public Health. (2022) 10:1047501. doi: 10.3389/fpubh.2022.1047501 36568802 PMC9773132

[37] Guo H. Risk identification and improvement strategies for BMI and physical fitness and health grade cross-classification: a cross-sectional study based on Chinese college students. Front Public Health. (2025) 13:1660686. doi: 10.3389/fpubh.2025.1660686 41246064 PMC12611645

[38] Gafni T Weinstein G Leonard D Barlow CE DeFina LF Pettee Gabriel K . Independent and joint associations of cardiorespiratory fitness and BMI with dementia risk: the Cooper Center Longitudinal Study. BMJ Open. (2023) 13:e075571. doi: 10.1136/bmjopen-2023-075571 38086580 PMC10729062

