# Supplementary material for: Trends and inequalities in physical fitness and BMI among Chinese college students: an analysis of national surveys between 2019 and 2023
Source: Front Public Health. 2026 Apr 20;14:1814894. doi: 10.3389/fpubh.2026.1814894 (PMC13136158; doi:10.3389/fpubh.2026.1814894)
Supplement: Supplementary file 1 [file Table_1.docx]

**Appendix**

Table S1. Parameters for median values and standard deviation of each physical fitness item for the reference population, 2019

| Age | Gender | FVC  (median ± sd) | SLJ  (median ± sd) | SAR  (median ± sd) | MS  (median ± sd) | SR  (median ± sd) | ER  (median ± sd) |
| --- | --- | --- | --- | --- | --- | --- | --- |
| 16 | Boys | 4366.5 ± 691.7 | 227.0 ± 21.6 | 10.7 ± 5.6 | 7.0 ± 7.1 | 7.1 ± 0.55 | 255.0 ± 31.5 |
| 16 | Girls | 3050.0 ± 503.9 | 174.0 ± 18.1 | 16.0 ± 5.7 | 45.0 ± 8.5 | 8.6 ± 0.69 | 243.0 ± 30.4 |
| 17 | Boys | 4177.0 ± 701.0 | 230.0 ± 22.2 | 11.4 ± 5.8 | 4.0 ± 7.2 | 7.1 ± 0.56 | 241.0 ± 27.8 |
| 17 | Girls | 2909.0 ± 486.5 | 174.0 ± 16.8 | 15.7 ± 6.3 | 42.0 ± 10.1 | 8.8 ± 0.64 | 234.0 ± 23.0 |
| 18 | Boys | 4161.0 ± 677.7 | 230.0 ± 21.2 | 11.7 ± 6.0 | 4.0 ± 6.7 | 7.1 ± 0.57 | 239.0 ± 24.1 |
| 18 | Girls | 2914.5 ± 490.4 | 175.0 ± 17.4 | 17.2 ± 5.9 | 42.0 ± 9.1 | 8.7 ± 0.69 | 229.0 ± 20.6 |
| 19 | Boys | 4234.0 ± 689.7 | 232.0 ± 21.9 | 11.1 ± 5.7 | 7.0 ± 7.8 | 6.9 ± 0.47 | 239.0 ± 23.9 |
| 19 | Girls | 2935.5 ± 487.8 | 178.0 ± 17.0 | 16.4 ± 6.0 | 48.0 ± 7.9 | 8.6 ± 0.61 | 231.0 ± 21.2 |
| 20 | Boys | 4410.0 ± 727.2 | 233.5 ± 23.5 | 12.9 ± 6.4 | 7.0 ± 8.3 | 7.0 ± 0.60 | 243.5 ± 31.7 |
| 20 | Girls | 2950.0 ± 466.6 | 176.0 ± 19.5 | 14.9 ± 6.2 | 45.5 ± 9.6 | 8.5 ± 0.67 | 232.0 ± 24.7 |
| 21 | Boys | 3881.0 ± 1033.9 | 218.0 ± 23.3 | 14.8 ± 7.2 | 4.0 ± 6.4 | 7.2 ± 0.47 | 249.0 ± 25.2 |
| 21 | Girls | 3116.0 ± 652.5 | 170.0 ± 24.0 | 19.1 ± 7.2 | 44.0 ± 10.0 | 8.8 ± 1.14 | 237.0 ± 30.6 |

Table S2. Median physical fitness index by year and nutritional status.

| Year | Nutritional status | Median_PFI | Q25 | Q75 |
| --- | --- | --- | --- | --- |
| 2019 | Normal | 0.44 | -1.77 | 2.65 |
| 2019 | Obesity | -2.36 | -4.43 | -0.62 |
| 2019 | Overweight | -0.78 | -2.79 | 1.3 |
| 2019 | Thinness | -0.38 | -2.34 | 1.64 |
| 2020 | Normal | -0.55 | -2.73 | 1.63 |
| 2020 | Obesity | -3.56 | -5.99 | -1.51 |
| 2020 | Overweight | -1.88 | -3.77 | 0.31 |
| 2020 | Thinness | -1.15 | -3.49 | 0.82 |
| 2021 | Normal | 0.04 | -2.17 | 2.38 |
| 2021 | Obesity | -3.22 | -5.75 | -1.15 |
| 2021 | Overweight | -1.36 | -3.35 | 0.9 |
| 2021 | Thinness | -0.83 | -3.09 | 1.3 |
| 2022 | Normal | -1.68 | -3.95 | 0.62 |
| 2022 | Obesity | -4.89 | -7.75 | -2.46 |
| 2022 | Overweight | -2.78 | -4.71 | -0.6 |
| 2022 | Thinness | -2.19 | -4.35 | 0.09 |
| 2023 | Normal | -0.46 | -2.71 | 1.88 |
| 2023 | Obesity | -3.59 | -6.56 | -1.34 |
| 2023 | Overweight | -1.44 | -3.5 | 0.68 |
| 2023 | Thinness | -1.36 | -3.59 | 0.81 |

Table S3. Annual distribution of PFI across gender and nutritional groups (2019–2023)

| Year | Gender | Nutritional status | Median_value | Q25 | Q75 |
| --- | --- | --- | --- | --- | --- |
| 2019 | Boys | Normal | 0.71 | -1.52 | 2.94 |
| 2019 | Boys | Obesity | -2.34 | -4.39 | -0.62 |
| 2019 | Boys | Overweight | -0.73 | -2.65 | 1.33 |
| 2019 | Boys | Thinness | -0.37 | -2.13 | 1.57 |
| 2019 | Girls | Normal | -0.02 | -2.24 | 2.15 |
| 2019 | Girls | Obesity | -2.72 | -4.65 | -0.63 |
| 2019 | Girls | Overweight | -1.03 | -3.38 | 1.22 |
| 2019 | Girls | Thinness | -0.39 | -2.7 | 1.76 |
| 2020 | Boys | Normal | -0.4 | -2.6 | 1.72 |
| 2020 | Boys | Obesity | -3.53 | -5.9 | -1.35 |
| 2020 | Boys | Overweight | -1.76 | -3.64 | 0.36 |
| 2020 | Boys | Thinness | -1.19 | -3.47 | 0.53 |
| 2020 | Girls | Normal | -0.84 | -2.99 | 1.46 |
| 2020 | Girls | Obesity | -4.08 | -6.36 | -2.46 |
| 2020 | Girls | Overweight | -2.44 | -4.48 | -0.15 |
| 2020 | Girls | Thinness | -1.09 | -3.54 | 1.08 |
| 2021 | Boys | Normal | 0.24 | -1.96 | 2.54 |
| 2021 | Boys | Obesity | -3.05 | -5.61 | -1.1 |
| 2021 | Boys | Overweight | -1.22 | -3.11 | 0.95 |
| 2021 | Boys | Thinness | -0.77 | -2.93 | 1.25 |
| 2021 | Girls | Normal | -0.32 | -2.56 | 2.08 |
| 2021 | Girls | Obesity | -4.02 | -6.35 | -1.72 |
| 2021 | Girls | Overweight | -1.89 | -4.09 | 0.61 |
| 2021 | Girls | Thinness | -0.91 | -3.21 | 1.32 |
| 2022 | Boys | Normal | -1.44 | -3.61 | 0.78 |
| 2022 | Boys | Obesity | -4.77 | -7.63 | -2.37 |
| 2022 | Boys | Overweight | -2.6 | -4.47 | -0.5 |
| 2022 | Boys | Thinness | -2.07 | -4.08 | 0.07 |
| 2022 | Girls | Normal | -2.24 | -4.77 | 0.3 |
| 2022 | Girls | Obesity | -5.34 | -8.07 | -3.04 |
| 2022 | Girls | Overweight | -3.58 | -5.98 | -1.12 |
| 2022 | Girls | Thinness | -2.31 | -4.73 | 0.09 |
| 2023 | Boys | Normal | -0.18 | -2.35 | 2.16 |
| 2023 | Boys | Obesity | -3.53 | -6.55 | -1.32 |
| 2023 | Boys | Overweight | -1.33 | -3.29 | 0.74 |
| 2023 | Boys | Thinness | -1.33 | -3.35 | 0.74 |
| 2023 | Girls | Normal | -1.09 | -3.28 | 1.19 |
| 2023 | Girls | Obesity | -4.14 | -6.66 | -1.89 |
| 2023 | Girls | Overweight | -2.49 | -4.55 | 0.15 |
| 2023 | Girls | Thinness | -1.44 | -3.96 | 1.03 |

Table S4. Temporal trends in PFI among Chinese college students across nutritional status groups, 2019–2023.

| **Year** | **Nutritional status** | **Variable** | **Median_PFI** | **Q25** | **Q75** |
| --- | --- | --- | --- | --- | --- |
| 2019 | Normal | ER_z | -0.1 | -0.63 | 0.48 |
| 2019 | Normal | FVC_z | -0.03 | -0.66 | 0.64 |
| 2019 | Normal | MS_z | 0.15 | -0.54 | 0.88 |
| 2019 | Normal | SAR_z | 0.02 | -0.65 | 0.73 |
| 2019 | Normal | SLJ_z | 0.11 | -0.54 | 0.74 |
| 2019 | Normal | SR_z | -0.08 | -0.64 | 0.52 |
| 2019 | Obesity | ER_z | 1.08 | 0.43 | 1.84 |
| 2019 | Obesity | FVC_z | 0.69 | -0.02 | 1.34 |
| 2019 | Obesity | MS_z | -0.6 | -0.9 | -0.42 |
| 2019 | Obesity | SAR_z | 0.05 | -0.7 | 0.76 |
| 2019 | Obesity | SLJ_z | -0.77 | -1.4 | -0.23 |
| 2019 | Obesity | SR_z | 0.74 | 0.18 | 1.45 |
| 2019 | Overweight | ER_z | 0.29 | -0.22 | 0.87 |
| 2019 | Overweight | FVC_z | 0.39 | -0.28 | 1.06 |
| 2019 | Overweight | MS_z | -0.39 | -0.6 | 0.42 |
| 2019 | Overweight | SAR_z | 0.12 | -0.6 | 0.82 |
| 2019 | Overweight | SLJ_z | -0.32 | -0.93 | 0.28 |
| 2019 | Overweight | SR_z | 0.29 | -0.35 | 0.91 |
| 2019 | Thinness | ER_z | -0.1 | -0.61 | 0.44 |
| 2019 | Thinness | FVC_z | -0.51 | -1.11 | 0.09 |
| 2019 | Thinness | MS_z | 0.14 | -0.47 | 0.79 |
| 2019 | Thinness | SAR_z | -0.29 | -0.9 | 0.47 |
| 2019 | Thinness | SLJ_z | 0.14 | -0.45 | 0.83 |
| 2019 | Thinness | SR_z | 0 | -0.54 | 0.54 |
| 2020 | Normal | ER_z | 0.17 | -0.43 | 0.85 |
| 2020 | Normal | FVC_z | 0.23 | -0.4 | 0.89 |
| 2020 | Normal | MS_z | -0.14 | -0.6 | 0.52 |
| 2020 | Normal | SAR_z | 0.15 | -0.56 | 0.87 |
| 2020 | Normal | SLJ_z | 0.24 | -0.33 | 0.88 |
| 2020 | Normal | SR_z | 0.87 | 0.3 | 1.42 |
| 2020 | Obesity | ER_z | 1.56 | 0.83 | 2.49 |
| 2020 | Obesity | FVC_z | 0.75 | 0.07 | 1.49 |
| 2020 | Obesity | MS_z | -0.6 | -0.9 | -0.56 |
| 2020 | Obesity | SAR_z | 0.07 | -0.61 | 0.98 |
| 2020 | Obesity | SLJ_z | -0.55 | -1.13 | 0 |
| 2020 | Obesity | SR_z | 1.78 | 1.06 | 2.44 |
| 2020 | Overweight | ER_z | 0.68 | 0.04 | 1.34 |
| 2020 | Overweight | FVC_z | 0.6 | -0.02 | 1.27 |
| 2020 | Overweight | MS_z | -0.56 | -0.77 | 0 |
| 2020 | Overweight | SAR_z | 0.2 | -0.52 | 0.89 |
| 2020 | Overweight | SLJ_z | -0.18 | -0.77 | 0.45 |
| 2020 | Overweight | SR_z | 1.25 | 0.7 | 1.91 |
| 2020 | Thinness | ER_z | 0.2 | -0.38 | 0.83 |
| 2020 | Thinness | FVC_z | -0.32 | -0.87 | 0.28 |
| 2020 | Thinness | MS_z | -0.13 | -0.64 | 0.56 |
| 2020 | Thinness | SAR_z | -0.1 | -0.8 | 0.64 |
| 2020 | Thinness | SLJ_z | 0.36 | -0.25 | 0.96 |
| 2020 | Thinness | SR_z | 0.89 | 0.29 | 1.42 |
| 2021 | Normal | ER_z | 0.19 | -0.46 | 0.92 |
| 2021 | Normal | FVC_z | -0.15 | -0.76 | 0.49 |
| 2021 | Normal | MS_z | 0.15 | -0.44 | 0.9 |
| 2021 | Normal | SAR_z | 0.38 | -0.33 | 1.08 |
| 2021 | Normal | SLJ_z | -0.05 | -0.67 | 0.61 |
| 2021 | Normal | SR_z | 0 | -0.58 | 0.58 |
| 2021 | Obesity | ER_z | 1.7 | 0.87 | 2.82 |
| 2021 | Obesity | FVC_z | 0.41 | -0.24 | 1.07 |
| 2021 | Obesity | MS_z | -0.56 | -0.77 | -0.24 |
| 2021 | Obesity | SAR_z | 0.35 | -0.42 | 1.05 |
| 2021 | Obesity | SLJ_z | -0.97 | -1.7 | -0.37 |
| 2021 | Obesity | SR_z | 0.85 | 0.18 | 1.48 |
| 2021 | Overweight | ER_z | 0.72 | 0.07 | 1.49 |
| 2021 | Overweight | FVC_z | 0.23 | -0.4 | 0.92 |
| 2021 | Overweight | MS_z | -0.3 | -0.6 | 0.43 |
| 2021 | Overweight | SAR_z | 0.4 | -0.25 | 1.1 |
| 2021 | Overweight | SLJ_z | -0.47 | -1.13 | 0.12 |
| 2021 | Overweight | SR_z | 0.36 | -0.21 | 1.06 |
| 2021 | Thinness | ER_z | 0.25 | -0.37 | 1.12 |
| 2021 | Thinness | FVC_z | -0.7 | -1.27 | -0.07 |
| 2021 | Thinness | MS_z | 0.15 | -0.43 | 0.88 |
| 2021 | Thinness | SAR_z | 0.14 | -0.62 | 0.85 |
| 2021 | Thinness | SLJ_z | 0 | -0.59 | 0.64 |
| 2021 | Thinness | SR_z | 0.15 | -0.53 | 0.71 |
| 2022 | Normal | ER_z | 0.53 | -0.24 | 1.49 |
| 2022 | Normal | FVC_z | -0.15 | -0.73 | 0.47 |
| 2022 | Normal | MS_z | -0.15 | -0.6 | 0.51 |
| 2022 | Normal | SAR_z | 0.12 | -0.61 | 0.8 |
| 2022 | Normal | SLJ_z | 0.05 | -0.64 | 0.66 |
| 2022 | Normal | SR_z | 0.83 | 0.21 | 1.4 |
| 2022 | Obesity | ER_z | 2.07 | 1.1 | 3.44 |
| 2022 | Obesity | FVC_z | 0.44 | -0.26 | 1.11 |
| 2022 | Obesity | MS_z | -0.6 | -0.85 | -0.56 |
| 2022 | Obesity | SAR_z | 0.14 | -0.62 | 0.9 |
| 2022 | Obesity | SLJ_z | -0.91 | -1.58 | -0.29 |
| 2022 | Obesity | SR_z | 1.74 | 1.07 | 2.44 |
| 2022 | Overweight | ER_z | 1.02 | 0.24 | 1.94 |
| 2022 | Overweight | FVC_z | 0.21 | -0.38 | 0.83 |
| 2022 | Overweight | MS_z | -0.56 | -0.73 | 0 |
| 2022 | Overweight | SAR_z | 0.18 | -0.47 | 0.9 |
| 2022 | Overweight | SLJ_z | -0.41 | -1.05 | 0.19 |
| 2022 | Overweight | SR_z | 1.18 | 0.53 | 1.83 |
| 2022 | Thinness | ER_z | 0.42 | -0.29 | 1.46 |
| 2022 | Thinness | FVC_z | -0.62 | -1.2 | -0.01 |
| 2022 | Thinness | MS_z | -0.14 | -0.6 | 0.47 |
| 2022 | Thinness | SAR_z | -0.08 | -0.79 | 0.62 |
| 2022 | Thinness | SLJ_z | 0.09 | -0.52 | 0.72 |
| 2022 | Thinness | SR_z | 0.83 | 0.29 | 1.39 |
| 2023 | Normal | ER_z | 0.38 | -0.33 | 1.24 |
| 2023 | Normal | FVC_z | -0.13 | -0.73 | 0.47 |
| 2023 | Normal | MS_z | 0.15 | -0.51 | 0.85 |
| 2023 | Normal | SAR_z | 0.24 | -0.49 | 0.95 |
| 2023 | Normal | SLJ_z | 0 | -0.66 | 0.66 |
| 2023 | Normal | SR_z | 0 | -0.52 | 0.7 |
| 2023 | Obesity | ER_z | 1.84 | 0.93 | 3.1 |
| 2023 | Obesity | FVC_z | 0.46 | -0.18 | 1.16 |
| 2023 | Obesity | MS_z | -0.6 | -0.85 | -0.3 |
| 2023 | Obesity | SAR_z | 0.14 | -0.61 | 0.9 |
| 2023 | Obesity | SLJ_z | -0.9 | -1.6 | -0.26 |
| 2023 | Obesity | SR_z | 0.85 | 0.17 | 1.7 |
| 2023 | Overweight | ER_z | 0.83 | 0.13 | 1.62 |
| 2023 | Overweight | FVC_z | 0.24 | -0.35 | 0.84 |
| 2023 | Overweight | MS_z | -0.39 | -0.62 | 0.31 |
| 2023 | Overweight | SAR_z | 0.3 | -0.42 | 1 |
| 2023 | Overweight | SLJ_z | -0.4 | -1 | 0.23 |
| 2023 | Overweight | SR_z | 0.36 | -0.21 | 1.07 |
| 2023 | Thinness | ER_z | 0.56 | -0.2 | 1.39 |
| 2023 | Thinness | FVC_z | -0.67 | -1.26 | -0.07 |
| 2023 | Thinness | MS_z | 0.15 | -0.55 | 0.83 |
| 2023 | Thinness | SAR_z | -0.03 | -0.78 | 0.73 |
| 2023 | Thinness | SLJ_z | 0.05 | -0.62 | 0.63 |
| 2023 | Thinness | SR_z | 0.17 | -0.42 | 0.83 |

Table S5. Changes in PFI of college students by birthplace, 2019–2023.

| Birthplace | Year_2019 | Year_2021 | Year_2023 | Change |
| --- | --- | --- | --- | --- |
| 上海市 | -0.1 | -0.39 | -1.13 | -1.02 |
| 云南省 | 0.77 | -0.05 | 0.19 | -0.58 |
| 内蒙古自治区 | -0.59 | -0.55 | -1.35 | -0.76 |
| 北京市 | 1.05 | 0.87 | 0.47 | -0.58 |
| 吉林省 | -1.44 | -1.41 | -1.92 | -0.47 |
| 四川省 | 0.37 | -0.44 | -0.46 | -0.83 |
| 天津市 | -0.19 | -0.61 | -0.9 | -0.71 |
| 宁夏回族自治区 | 0.28 | -1.4 | -1.47 | -1.75 |
| 安徽省 | -0.22 | -0.43 | -0.91 | -0.69 |
| 山东省 | -0.28 | -1.04 | -1.63 | -1.35 |
| 山西省 | -0.14 | -0.79 | -0.85 | -0.71 |
| 广东省 | 0.09 | -0.24 | -0.76 | -0.86 |
| 广西壮族自治区 | 0.06 | -0.17 | -0.8 | -0.86 |
| 新疆维吾尔自治区 | -0.15 | -0.83 | -1.2 | -1.05 |
| 江苏省 | -0.35 | -0.85 | -1 | -0.65 |
| 江西省 | -0.05 | 0.15 | -1.28 | -1.23 |
| 河北省 | -0.44 | -1.21 | -1.68 | -1.25 |
| 河南省 | 0.28 | -0.09 | -1.02 | -1.3 |
| 浙江省 | 0.64 | 0.29 | -0.2 | -0.84 |
| 海南省 | -1.03 | -0.8 | -1.46 | -0.43 |
| 湖北省 | -0.51 | -0.99 | -1.56 | -1.05 |
| 湖南省 | -0.08 | -0.68 | -1.18 | -1.1 |
| 甘肃省 | -0.23 | -1.21 | -1.28 | -1.06 |
| 福建省 | -0.65 | -0.99 | -1.91 | -1.26 |
| 西藏自治区 | -0.98 | -0.97 | -2.15 | -1.17 |
| 贵州省 | -0.47 | -1 | -1.48 | -1.01 |
| 辽宁省 | -0.11 | -1.06 | -1.39 | -1.28 |
| 重庆市 | -0.04 | -0.48 | -0.64 | -0.59 |
| 陕西省 | -0.53 | -0.74 | -1.44 | -0.92 |
| 青海省 | -0.24 | -0.69 | -1.43 | -1.19 |
| 黑龙江省 | -1.28 | -1.94 | -2.43 | -1.15 |

Table S6. Provincial changes in normal nutritional status among college students (2019–2023).

| Birthplace | prop2019 | prop2020 | prop2021 | prop2022 | prop2023 |
| --- | --- | --- | --- | --- | --- |
| 上海市 | 0.70 | 0.65 | 0.66 | 0.63 | 0.63 |
| 云南省 | 0.71 | 0.66 | 0.65 | 0.66 | 0.62 |
| 内蒙古自治区 | 0.59 | 0.58 | 0.56 | 0.55 | 0.54 |
| 北京市 | 0.64 | 0.66 | 0.65 | 0.67 | 0.67 |
| 吉林省 | 0.52 | 0.57 | 0.55 | 0.58 | 0.57 |
| 四川省 | 0.67 | 0.65 | 0.66 | 0.65 | 0.64 |
| 天津市 | 0.58 | 0.60 | 0.65 | 0.65 | 0.64 |
| 宁夏回族自治区 | 0.68 | 0.62 | 0.67 | 0.61 | 0.69 |
| 安徽省 | 0.65 | 0.63 | 0.62 | 0.61 | 0.63 |
| 山东省 | 0.62 | 0.62 | 0.59 | 0.55 | 0.55 |
| 山西省 | 0.64 | 0.63 | 0.62 | 0.60 | 0.62 |
| 广东省 | 0.66 | 0.68 | 0.66 | 0.67 | 0.68 |
| 广西壮族自治区 | 0.70 | 0.68 | 0.71 | 0.68 | 0.70 |
| 新疆维吾尔自治区 | 0.66 | 0.64 | 0.62 | 0.62 | 0.62 |
| 江苏省 | 0.68 | 0.65 | 0.64 | 0.59 | 0.60 |
| 江西省 | 0.73 | 0.73 | 0.70 | 0.70 | 0.67 |
| 河北省 | 0.63 | 0.59 | 0.62 | 0.59 | 0.57 |
| 河南省 | 0.67 | 0.67 | 0.65 | 0.61 | 0.60 |
| 浙江省 | 0.66 | 0.71 | 0.70 | 0.69 | 0.71 |
| 海南省 | 0.63 | 0.68 | 0.70 | 0.73 | 0.69 |
| 湖北省 | 0.66 | 0.62 | 0.62 | 0.60 | 0.63 |
| 湖南省 | 0.66 | 0.68 | 0.64 | 0.63 | 0.63 |
| 甘肃省 | 0.65 | 0.65 | 0.67 | 0.63 | 0.59 |
| 福建省 | 0.65 | 0.61 | 0.68 | 0.61 | 0.66 |
| 西藏自治区 | 0.82 | 0.70 | 0.70 | 0.50 | 0.57 |
| 贵州省 | 0.65 | 0.66 | 0.62 | 0.61 | 0.64 |
| 辽宁省 | 0.58 | 0.57 | 0.57 | 0.56 | 0.57 |
| 重庆市 | 0.72 | 0.66 | 0.66 | 0.64 | 0.65 |
| 陕西省 | 0.67 | 0.61 | 0.63 | 0.60 | 0.63 |
| 青海省 | 0.66 | 0.66 | 0.71 | 0.69 | 0.61 |
| 黑龙江省 | 0.55 | 0.60 | 0.58 | 0.59 | 0.57 |


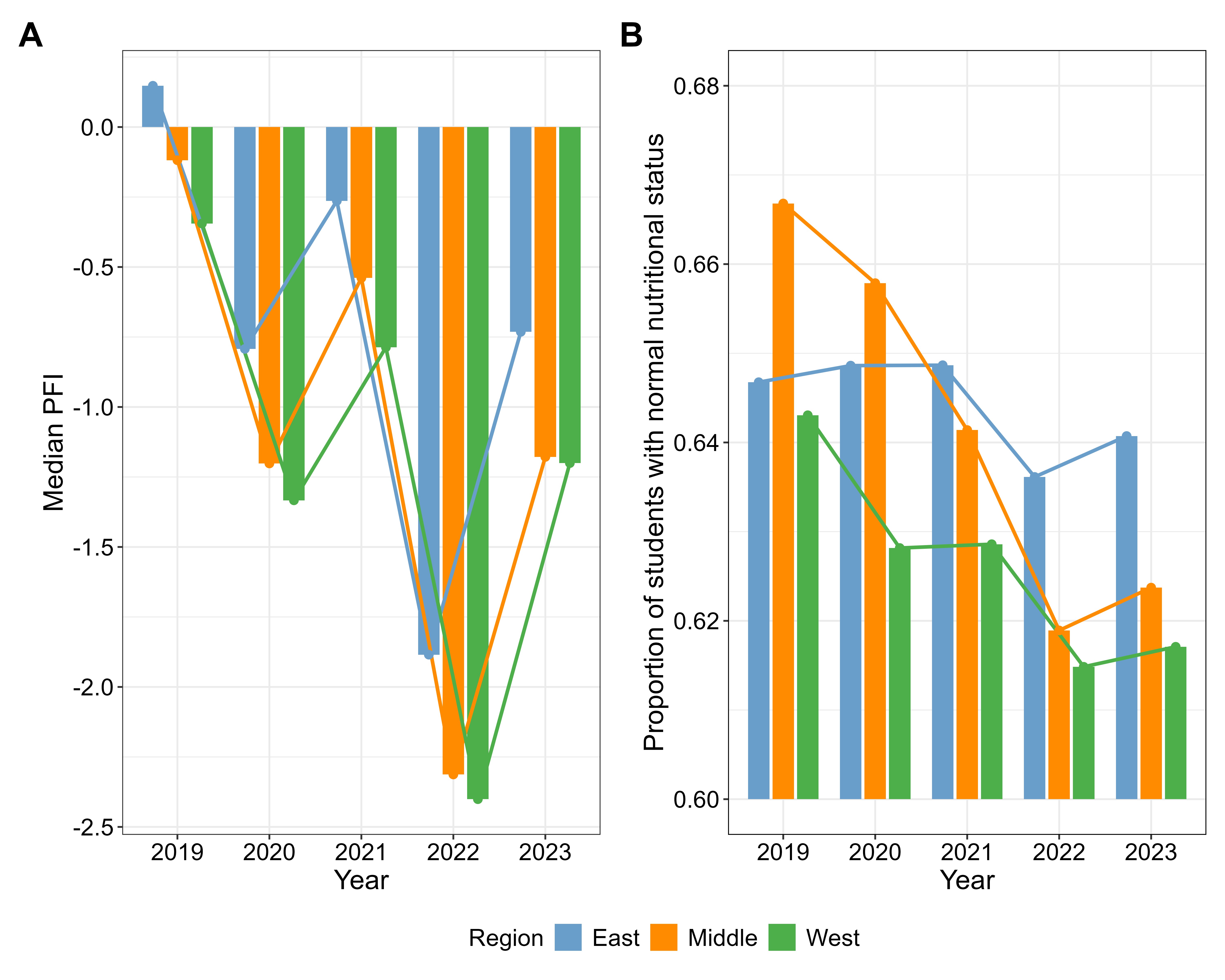


Figure S1. Temporal trends in physical fitness and nutritional status among Chinese college students (2019–2023). A Changes in the PFI of college students across different regions from 2019 to 2023.

B Changes in the proportion of students with normal nutritional status across regions from 2019 to 2023.

Table S7. Regression coefficients for the restricted cubic spline models of BMI and PFI.

| Group | Term | Beta | SE | t_value | P_value |
| --- | --- | --- | --- | --- | --- |
| 2019 | BMI | 0.491 | 0.048 | 10.300 | 0.000 |
| 2019 | BMI' | -2.272 | 0.226 | -10.061 | 0.000 |
| 2019 | BMI'' | 4.806 | 0.600 | 8.004 | 0.000 |
| 2020 | BMI | 0.465 | 0.048 | 9.711 | 0.000 |
| 2020 | BMI' | -2.102 | 0.221 | -9.492 | 0.000 |
| 2020 | BMI'' | 4.318 | 0.588 | 7.348 | 0.000 |
| 2021 | BMI | 0.578 | 0.046 | 12.617 | 0.000 |
| 2021 | BMI' | -2.647 | 0.224 | -11.809 | 0.000 |
| 2021 | BMI'' | 5.404 | 0.584 | 9.249 | 0.000 |
| 2022 | BMI | 0.415 | 0.047 | 8.840 | 0.000 |
| 2022 | BMI' | -1.803 | 0.226 | -7.981 | 0.000 |
| 2022 | BMI'' | 3.220 | 0.588 | 5.479 | 0.000 |
| 2023 | BMI | 0.495 | 0.044 | 11.259 | 0.000 |
| 2023 | BMI' | -2.297 | 0.220 | -10.430 | 0.000 |
| 2023 | BMI'' | 4.488 | 0.574 | 7.819 | 0.000 |
| Overall | BMI | 0.472 | 0.021 | 22.308 | 0.000 |
| Overall | BMI' | -2.186 | 0.102 | -21.450 | 0.000 |
| Overall | BMI'' | 4.362 | 0.268 | 16.300 | 0.000 |

Table S8. Estimated BMI effects on physical fitness components of college students (2019–2023).

| Outcome | Term | Beta | SE | t_value | P_value |
| --- | --- | --- | --- | --- | --- |
| FVC_z | BMI | 0.167 | 0.006 | 29.079 | 0.000 |
| FVC_z | BMI' | -0.161 | 0.028 | -5.834 | 0.000 |
| FVC_z | BMI'' | 0.184 | 0.072 | 2.547 | 0.011 |
| SLJ_z | BMI | 0.032 | 0.006 | 5.313 | 0.000 |
| SLJ_z | BMI' | -0.454 | 0.029 | -15.730 | 0.000 |
| SLJ_z | BMI'' | 1.048 | 0.076 | 13.827 | 0.000 |
| SAR_z | BMI | 0.104 | 0.006 | 16.853 | 0.000 |
| SAR_z | BMI' | -0.256 | 0.030 | -8.640 | 0.000 |
| SAR_z | BMI'' | 0.511 | 0.078 | 6.572 | 0.000 |
| MS_z | BMI | 0.063 | 0.006 | 11.245 | 0.000 |
| MS_z | BMI' | -0.590 | 0.027 | -21.753 | 0.000 |
| MS_z | BMI'' | 1.452 | 0.071 | 20.384 | 0.000 |
| SR_z | BMI | -0.046 | 0.006 | -7.437 | 0.000 |
| SR_z | BMI' | 0.420 | 0.030 | 14.067 | 0.000 |
| SR_z | BMI'' | -0.895 | 0.078 | -11.419 | 0.000 |
| ER_z | BMI | -0.061 | 0.007 | -8.133 | 0.000 |
| ER_z | BMI' | 0.305 | 0.036 | 8.501 | 0.000 |
| ER_z | BMI'' | -0.271 | 0.094 | -2.879 | 0.004 |
